# Effects of COVID-19 lockdown in Milan urban and Rome suburban acoustic environments: Anomalous noise events and intermittency ratioa)

**DOI:** 10.1121/10.0009783

**Published:** 2022-03-11

**Authors:** Francesc Alías, Rosa Ma. Alsina-Pagès

**Affiliations:** GTM - Grup de Recerca en Tecnologies Mèdia, La Salle - Universitat Ramon Llull, C/Quatre Camins, 30, 08022, Barcelona, Spain

## Abstract

The COVID-19 pandemic affected the acoustic environment worldwide, entailing relevant reductions of equivalent noise levels (*L_Aeq_*) during this exceptional period. In the context of the LIFE+ DYNAMAP project, two wireless acoustic sensor networks were deployed in Milan and Rome. Taking advantage of the built-in identification of anomalous noise events (ANE) in the sensors, this work analyses the effects of the COVID-19 lockdown in both urban and suburban acoustic environments from January to June 2020, considering the distribution of ANEs and the intermittency ratio (IR) as an indicator of the impact of noise on population. The results show statistically significant increments of ANEs in Rome during the lockdown, mainly on weekends, and especially at night, despite the significant decrease in salient events. Differently, ANEs decrease during the lockdown in Milan, mostly at daytime, as a result of population confinement. Although the IR increases in several urban locations, most sensed locations show a relevant decrease in IR during the confinement, which represents a noteworthy reduction of the negative impact of noise in the population of both cities. During the post-lockdown period, all the scores start to return to those observed in the pre-lockdown, but still remaining higher than in 2019.

## I. INTRODUCTION

The COVID-19 pandemic started in Wuhan, China in December 2019. The World Health Organization declared it as a public health emergency on January 30, 2020 (Jee, 2020). Authorities from countries worldwide started to define different containment measures, with the final goal of saving citizens' lives by preventing them from virus contagion. These measures brought a decrease in anthropic noise during this period, as only essential activities were permitted, while most countries also banned social events.

These measures had a huge impact on environmental noise pollution and on the soundscape of cities worldwide, mainly in urban environments (Aletta *et al.*, 2020b). This exceptional situation posed new noise research challenges, even requiring the definition of novel terms and taxonomies (Asensio *et al.*, 2020a). Among other metrics, it considers the intermittency ratio (IR) (Wunderli *et al.*, 2016) as an indicator of the noise impact on people (Brink *et al.*, 2016; Héritier *et al.*, 2018), since it is correlated with higher risks of cardiovascular diseases and annoyance (Tong and Kang, 2021). Several types of environmental noise sources significantly decreased during this period, such as the main noise source in cities, road traffic noise (RTN) (Aletta *et al.*, 2020a; Asensio *et al.*, 2020b). However, changes in railway, port, airport, and industry noise were also observed (Aletta *et al.*, 2020a), as well as relevant leisure noise reductions in those areas with restaurants and bars (Alsina-Pagès *et al.*, 2021), making it possible to hear more nature sounds that were previously masked by transport-related noise (BruitParif, 2020; Zambon *et al.*, 2021).

Most of this research has been conducted all around the world by means of wireless acoustic sensor networks (WASNs), making the comparison with previous measurements possible by computing *L_Aeq_*, or long-exposure evaluations, such as *L_day_*, *L_night_*, and *L_den_* [e.g., see Aletta *et al.*, 2020a; Alsina-Pagès *et al.*, 2020; Asensio *et al.*, 2020b]. In France, the permanent sensor networks deployed across the Île-de-France region showed significant soundscape variations at many locations in Paris and its surroundings (BruitParif, 2020), obtaining the deepest drops in terms of *L_Aeq_* in the sensors installed in RTN environments, but also in leisure areas. In Dublin (Ireland), a 12-sensor network was used to analyse the changes in the acoustic environment, deducing a clear decrease in road traffic and air pollution during the lockdown in all the monitoring stations (Basu *et al.*, 2021). In the city of Montreal (Canada), the soundscape transformation during the lockdown was studied in three locations, using continuous data gathered before, during, and after the lockdown in the framework of the *Sounds in the City* project (Steele *et al.*, 2020). The analysis revealed noise reductions of 6–7 dBA during the lockdown stage. These levels were gradually recovered as restrictions were relaxed after the severe closure of the city (Steele and Guastavino, 2021). In the city of Buenos Aires (Argentina), the analysis was conducted on big roads with heavy traffic (Said *et al.*, 2020), showing a clear decrease in noise levels mainly at night. In Stockholm (Sweden), the researchers followed an alternative approach, using a 1-yr noise recording campaign from the city center (Rumpler *et al.*, 2020), obtaining noise drops of around 3 dB. Rio de Janeiro (Brazil) had their highest level of social isolation in July 2020, and several measurements of *L_Aeq_* were conducted in 12 locations of the city (Gevú *et al.*, 2021). The highest noise drop was obtained due to outdoor human activity reduction (e.g., people talking, markets, loudspeakers, etc.), followed by the decrease in RTN levels. In Spain, several studies have also been conducted by means of WASNs. The data collected by the 31-acoustic sensor network installed in Madrid was used to study the lockdown equivalent noise levels across the city in 2020 (Asensio *et al.*, 2020b), showing a clear decrease in the overall noise levels, especially in the traffic dominated areas, with higher reduction in the weekends, probably due to the lack of commercial activity. By means of the Barcelona Noise Monitoring Network (Bonet-Solà *et al.*, 2021), a drastic reduction of *L_Aeq_* in nightlife areas of the city was observed, together with a moderate-to-high change in commercial and restaurant areas, and a small decrease in noise levels in dense traffic areas. Moreover, in Girona (Alsina-Pagès *et al.*, 2021), and also using the data from a small WASN composed of eight sensors, the study again showed a clear decrease in noise in the street, coming from any noise source. The authors also highlight that the largest differences on *L_Aeq_* values were found on those locations containing leisure noise, being even higher than those where RTN was the main source. Finally, in Italy, a clear decrease in *L_Aeq_* levels was observed in Milan and Rome during the lockdown by means of the two WASNs deployed by the LIFE+ DYNAMAP project (Sevillano *et al.*, 2016) due to important road traffic flow reductions (Alsina-Pagès *et al.*, 2020; Zambon *et al.*, 2021). In Alsina-Pagès *et al.* (2020), the presence of non-traffic noise events, denoted as anomalous noise events (ANE) (Sevillano *et al.*, 2016) (e.g., trains, trams, sirens, birdsongs, or people talking) was also analysed, due to their built-in identification in the WASN nodes. However, that analysis only considered one weekday and one weekend day in March 2020. This work studies the effects of the COVID-19 knockdown in both urban and suburban acoustic environments by analysing the distribution of ANEs and the impact on noise on population based on IR variations, both from January to June 2020 with respect to 2019, which covers pre-lockdown (PreL), lockdown (Lock), and post-lockdown (PostL) periods.

This paper is structured as follows. Section II describes the algorithm that detects ANEs in real time. Next, Sec. III details the data collection process as well as the Italian lockdown periods. Section IV presents the results of the conducted experiments. Finally, Sec. V discusses the main conclusions obtained from this research and presents future work.

## II. ANOMALOUS NOISE EVENT DETECTOR

**FIG. 1.**
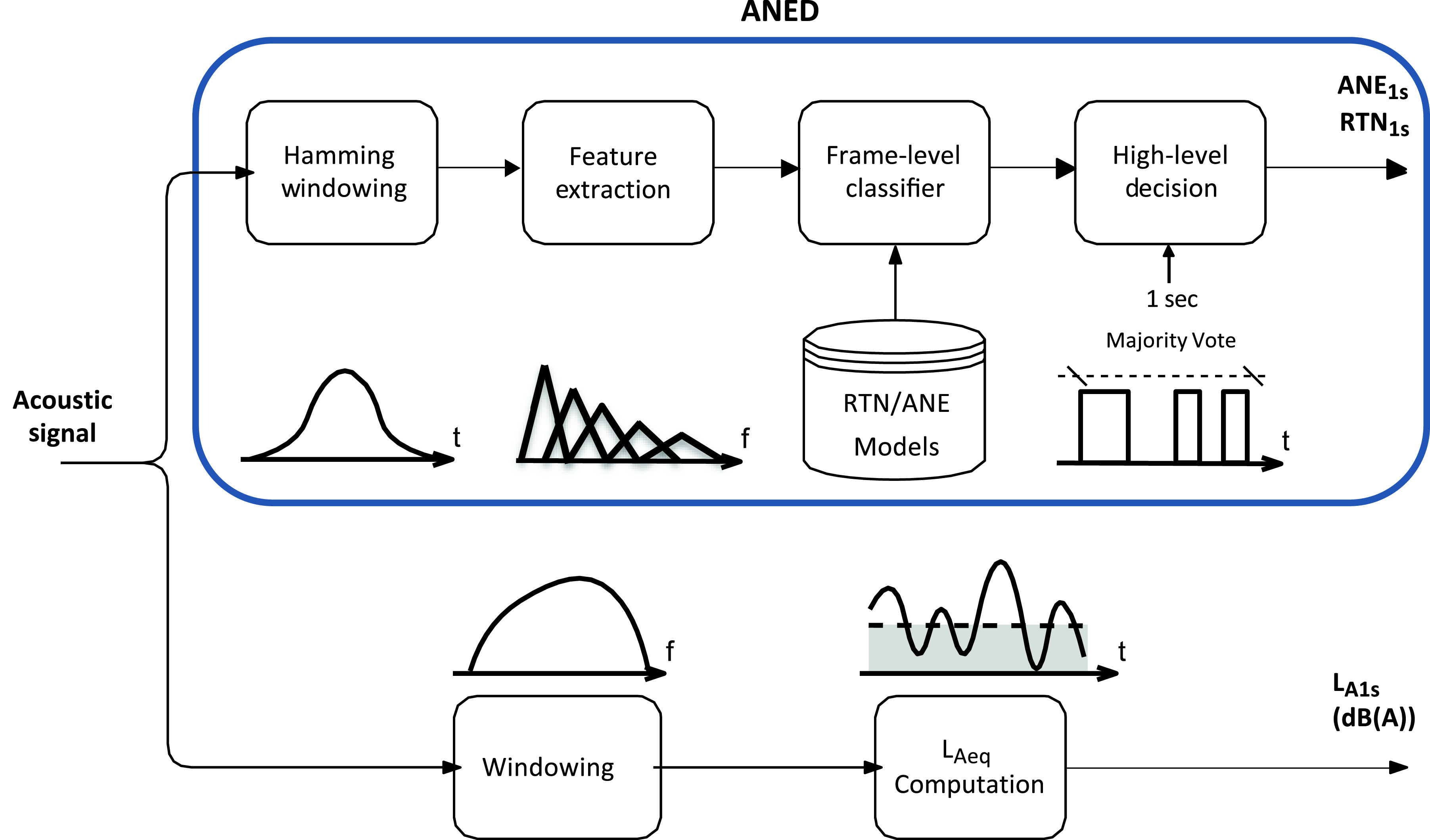
(Color online) Block diagram of the processes run by acoustic sensor, including the ANED algorithm and the computation of *L_Aeq_* every second from the input acoustic signal.

According to the project specifications, the anomalous noise events detector (ANED) is aimed at detecting the presence of any noise source not coming from vehicles' engines or from the regular interaction of their tires with the pavement (Sevillano *et al.*, 2016). The ANED labels the input audio captured by the acoustic sensor as RTN or ANE every second (Socoró *et al.*, 2017). This label is sent together with the *L_Aeq_* and also computed at the 1-s level ( $L_{A1s}$) (see Fig. 1) to the central platform to avoid biasing the dynamic update of the precomputed static RTN maps due to the presence of ANEs (Alías *et al.*, 2020a; Orga *et al.*, 2017).

The top branch of Fig. 1 shows the main blocks of the ANED pipeline. The process begins with 30 ms Hamming windowing of the input acoustic signal, followed by audio feature extraction based on the computation of Mel frequency cepstral coefficients (Mermelstein, 1976). This spectral parameterization is introduced into a two-class classifier based on Gaussian mixture models, which was trained using two acoustic datasets collected in late 2017 through the deployed WASNs, totalling more than 150 h of labelled data for each one (see Alsina-Pagès *et al.*, 2019; Alías *et al.*, 2020b for further details). The last stage of the algorithm corresponds to a high-level decision based on the majority voting of the frame-level binary decisions of the classifier. The current version of the ANED algorithm was implemented on both networks in April 2018 after training the algorithm with labelled WASN-based acoustic datasets collected from both acoustic environments (Alías *et al.*, 2020a; Alsina-Pagès *et al.*, 2019). Moreover, the ANED algorithm can also be used as a tool for analysing the changes on the environmental noise at both monitored from Milan and Rome, through the detection of ANEs, e.g., by describing the variation of their occurrences between day and night (Alías *et al.*, 2020b).

## III. DATA COLLECTION AND LOCKDOWN PERIODS

The DYNAMAP noise monitoring system is based on the Noisemote^®^ platform, which records historical data from all the sensors of both WASNs (from $L_{A1s}$ and ANED labels every second to *L_den_* values daily). In Rome, the sensors are installed in the portals of A90 motorway on different types of roads: single roads, crossings, nearby railways, and multiple connections (Bellucci *et al.*, 2017). In Milan, the WASN is deployed across District 9; a densely populated area. The sensors are placed on the façades of public buildings located in several street typologies (Zambon *et al.*, 2017). To analyse the distribution of ANEs, Noisemote^®^ data at the 1-s level have been gathered from January 1 to June 25 from 2020 and 2019, respectively. In particular, 20 out of the 24 sensors deployed in Milan and 11 out of the 19 nodes installed in Rome have been considered. The discarded nodes presented significant periods with non-available data (e.g., several days or a week) due to different technical problems. As a result, around 4.200 h of data per sensor and year are considered in the subsequent analyses, after removing these nodes following Alsina-Pagès *et al.* (2020) and Zambon *et al.* (2021).

Regarding the COVID-19 outbreak, Italy stated the first confirmed case on January 31, 2020 (Alsina-Pagès *et al.*, 2020). After its spread throughout the country, the Italian authorities declared the lockdown on March 10, banning almost all non-essential activities and making citizens stay at home by means of a National Decree. On May 4, the lockdown was partially lightened, and several outdoor activities reappeared gradually. During this post-lockdown period, museums, libraries, commerce, and later hairdressers, restaurants, and bars were reopened. Only schools openings were postponed until September. Nevertheless, several jobs were kept working from home, both from administration and private companies.

In order to group the different phases and specific measures implemented either in Rome and Milan, we consider three main periods around the Italian COVID-19 outbreak from January 1 to June 25 in 2020 (see Table I): (i) pre-lockdown (weeks 1 to 10), (ii) lockdown (weeks 11 to 18), and (iii) post-lockdown (weeks 19 to 25); the same time frame evaluated in Alsina-Pagès *et al.* (2020) and Zambon *et al.* (2021).

**TABLE I.** Dates of the considered analysis periods around the Italian COVID-19 lockdown during 2020.

Period	Dates	Weeks
Pre-lockdown	01/10/2020–08/03/2020	1–10
Lockdown	09/03/2020–03/05/2020	11–18
Post-lockdown	04/05/2020–21/06/2020	19–25

Finally, it is worth mentioning that although the ANED is not designed to determine the noise source, informed estimates can be made based on the characteristics of each acoustic environment, considering the restrictions applied in each period together with the meteorological data provided by the Italian Regional Agency for Environmental Protection (ARPA).

## IV. EXPERIMENTS AND RESULTS

This section describes the results of the conducted experiments for the Milan urban and Rome suburban acoustic environments, comparing the distribution of ANEs detected by the ANED together with the IR indicator from January to June 2020 with respect to 2019, differentiating weekdays from weekend days (Alsina-Pagès *et al.*, 2020; Asensio *et al.*, 2020b). The analyses are conducted at two levels: (i) on a period-basis to study the evolution of the ANEs within each period of interest, whose differences are statistically evaluated using the Mann-Whitney U Test (MW-U) (Mann and Whitney, 1947), and (ii) on an hour-per-weekly basis to study the distribution of the ANEs with respect to the salient events along the day, either during daytime (6:00–22:00 h) or at night (22:00–6:00 h) (Brambilla *et al.*, 2020).

### A. Period-based analysis

This section describes the results of the period-based analysis conducted in the areas of interest in terms of the distribution of events and variation of IR, differentiating weekdays from weekend days.

In order to analyse the distribution of anomalous events, the average percentage of ANEs detected per day is computed following Eq. (1). The result is subsequently accumulated and averaged every week, differentiating weekdays from weekend days to obtain the respective averaged ANE (%) for each period and year.

$$ANE^{d}=\frac{1}{N^{d}}\sum_{i=1}^{N^{d}}\#ANE_{1s}^{d},$$ (1)

where $\#ANE_{1s}^{d}$ represents the number of ANEs detected by the ANED throughout day *d* in all sensors per area at the second level, and *N^d^* denotes the total number of ANED output labels collected for that day (this value can slightly vary in some cases due to punctual technical problems derived from the communication between the sensor and the central platform).

To evaluate the impact of salient events along the day, the IR (%) (Wunderli *et al.*, 2016) is computed as:

$$IR=\frac{10^{0.1L_{\mathrm{Aeq},T,\mathrm{Events}}}}{10^{0.1L_{\mathrm{Aeq},T,\mathrm{Total}}}}100,$$ (2)

where $L_{\mathrm{Aeq},T,\mathrm{Total}}$ represents the total A-weighted equivalent noise level and $L_{\mathrm{Aeq},T,\mathrm{Event}}$ the corresponding value of the events surpassing in 3 dBs the $L_{\mathrm{Aeq},T,\mathrm{Total}}$ (Wunderli *et al.*, 2016) for T=1h. The IR (%) per period is obtained by averaging the result per weekdays or weekend days for each year and period of interest.

#### 1. Milan urban area

**FIG. 2.**
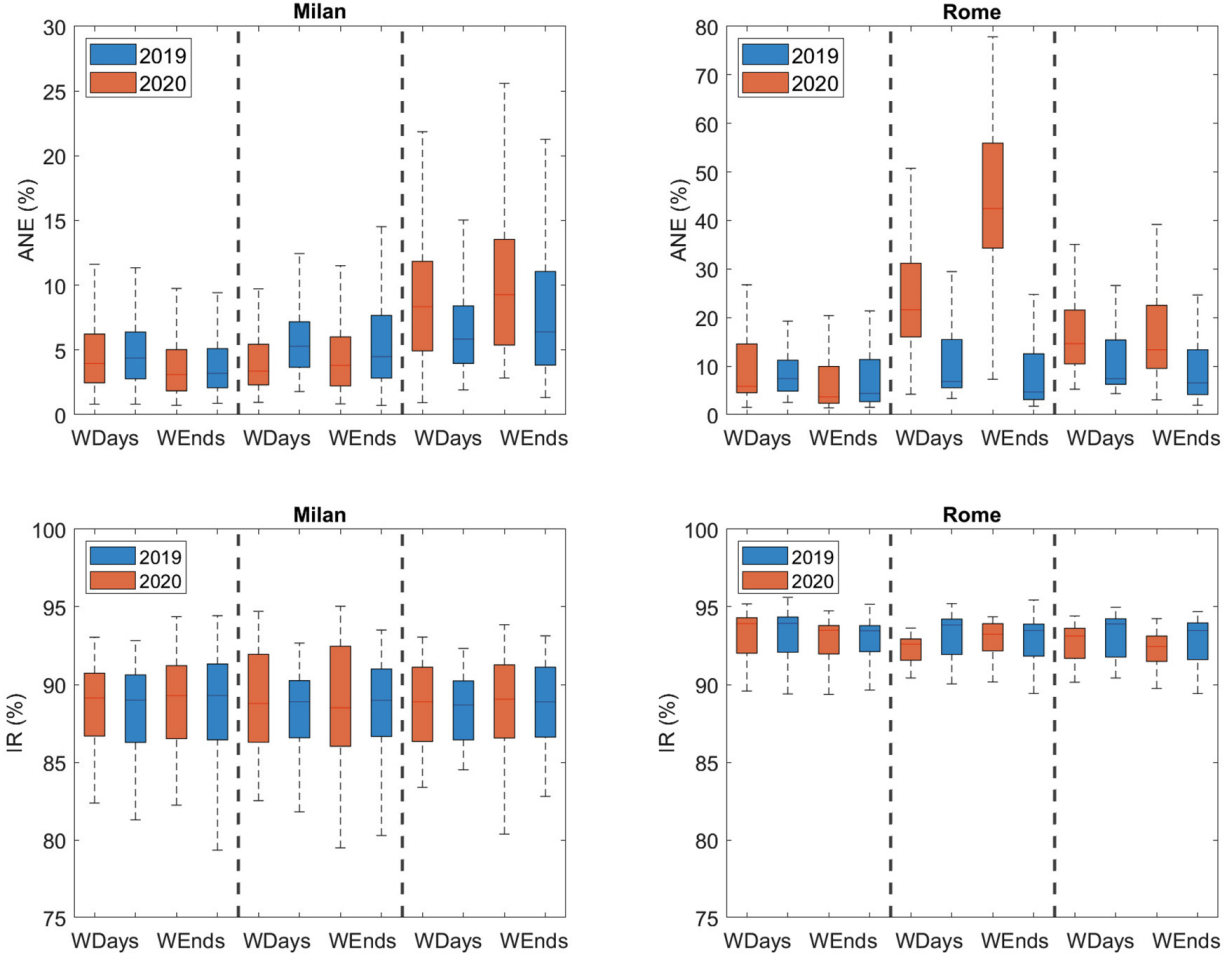
(Color online) Boxplot distributions of ANEs (%) and IR (%) in Milan and Rome on weekdays (WDays) and weekends (WEnds) per period in 2020 compared to 2019. The vertical dashed black lines group, from left to right, pre-lockdown, lockdown, and post-lockdown distributions, differentiating weekdays from weekend days within each period.

Figure 2(a) represents the ANE (%) distributions in Milan for the three periods in 2020 with respect to 2019, for weekdays and weekends. As it can be observed from the figure and the leftmost part of Table II, the ANE distributions are almost the same during the weekdays (mean: 5.02% in 2020 and 5.03% in 2019) and very similar on weekends (mean: 3.92% in 2020 and 4.40% in 2019), which is confirmed by the results of the MW-U test that state no statistically significant differences (*p* = 0.326 and *p* = 0.183 for weekdays and weekends, respectively). Regarding the lockdown, a relevant reduction of presence of the events is observed both during weekdays (mean: from 6.20% in 2019 to 4.53% in 2020) and weekends (mean: from 5.84% in 2019 to 4.66% in 2020); both differences are statistically significant (with *p* < 0.05). Finally, the post-lockdown presents a different behaviour, as the percentage of ANEs detected in 2020 is higher than in 2019 both on weekdays (rising their mean from 7.59%–9.48%) and at weekends (increasing their mean from 9.85%–10.18%). These differences are also statistically significant according to the MW-U test.

**TABLE II.** Mean ANE (%) and Salients (%) between 2020 and 2019 in Milan and Rome during weekdays and weekends per period, and mean differences in $\Delta ANE^{h}$ (%) and Δ Salients (%) along the day (6:00–22:00 h) and at night (22:00–6:00 h), denoted as ΔDay and ΔNight, respectively.

	Milan urban area								Rome suburban area							
	Weekdays				Weekends				Weekdays				Weekends			
Period	2020	2019	ΔDay	ΔNight	2020	2019	ΔDay	ΔNight	2020	2019	ΔDay	ΔNight	2020	2019	ΔDay	ΔNight
PreL ANEs	5.02	5.03	0.20	−0.39	3.92	4.40	−1.18	0.92	9.23	9.24	−0.70	1.28	7.06	8.54	−1.97	−0.41
Lock ANEs	4.53	6.20	−2.01	−0.99	4.66	5.84	−1.76	−0.03	24.86	10.49	13.13	16.45	44.38	8.34	34.84	37.20
PostL ANEs	9.48	7.59	0.55	4.24	10.18	9.85	0.24	−0.18	16.57	11.09	2.99	10.47	16.05	10.02	2.10	14.04
PreL Salients	36.88	37.34	−0.35	−0.94	34.20	35.13	−1.11	−0.59	67.27	67.69	−0.69	−0.13	66.25	67.16	−0.88	−0.96
Lock Salients	26.07	36.86	−13.34	−5.60	23.06	34.30	−14.48	−4.74	46.98	67.80	−24.27	−13.90	32.32	67.30	−40.10	−24.73
PostL Salients	33.40	37.36	−4.90	−2.08	30.69	34.73	−4.04	−4.04	61.21	68.61	−7.85	−6.49	57.74	68.47	−10.02	−12.13

Regarding the IR (%) distributions depicted in Fig. 2(c), no significant differences were found within any of the three periods of interest (i.e., MW-U test with *p* > 0.05), presenting very similar mean values around 88%–89%, as it can be observed in Table III. However, it is notable that higher deviation of the 2020 distributions during the lockdown compared to 2019 (increasing from 2.27%–3.23% on weekdays and from 2.78%–3.81% on weekends), which is also noticeable in the post-lockdown period (increasing from 2.14%–2.54% on weekdays and from 2.68%–3.01% on weekends), which denotes a larger variability on the IR in the urban acoustic environments during these periods.

**TABLE III.** Mean values ( ±σ) of IR (%) between 2020 and 2019 in Milan and Rome during weekdays and weekends per period.

	Milan urban area				Rome suburban area			
	Weekdays		Weekends		Weekdays		Weekends	
Period	2020	2019	2020	2019	2020	2019	2020	2019
PreL IR	88.72 (± 2.37)	88.51 (± 2.50)	88.88 (± 2.84)	88.83 (± 3.01)	93.26 (± 1.36)	93.31 (± 1.52)	92.85 (± 1.36)	92.91 (± 1.57)
Lock IR	89.15 (± 3.23)	88.62 (± 2.27)	88.86 (± 3.81)	88.80 (± 2.78)	92.34 (± 0.84)	93.23 (± 1.40)	92.94 (± 1.18)	92.95 (± 1.46)
PostL IR	88.75 (± 2.54)	88.65 (± 2.14)	88.77 (± 3.01)	88.78 (± 2.68)	92.68 (± 1.19)	93.18 (± 1.40)	92.25 (± 1.40)	92.88 (± 1.48)

#### 2. Rome suburban area

Figure 2(b) depicts the distributions of ANE (%) corresponding to Rome. In this case, the increment of the presence of ANEs during the lockdown with respect to the pre-lockdown period is very evident. This observation is confirmed by the values shown in the rightmost part of Table II, where the ANE percentages remain very similar in weeks 1–10, both during the weekdays (mean: 9.23% in 2020 and 9.24% in 2019) and at weekends (mean: 7.06% in 2020 and 8.54% in 2019). The MW-U tests statistically confirm these pair-based similarities (with *p* = 0.5162 and *p* = 0.2052 for weekdays and weekends, respectively). During the lockdown, a very significant increment of ANE is found in the suburban area during weekdays (mean: from 10.49% in 2019 to 24.85% in 2020) but especially at weekends, where the mean percentage of ANEs rises from 8.34% in 2019 to 44.38% in 2020. Again, these increments are statistically significant according to the results of the MW-U test. Finally, during the post-lockdown, the ANE (%) from 2020 (mean: 16.57% on weekdays and 16.05% at weekends) starts to return to the reference values in 2019 (mean: 11.09% on weekdays and 10.02% at weekends), but still being significantly larger (MW-U with *p* < 0.05).

Moreover, Fig. 2(d) depicts the corresponding IR (%) distributions, while Table III shows their mean plus standard deviation values. As already observed in Milan, no significant differences are found in the pre-lockdown period, according to the MW-U test (with *p* < 0.05) (*p* = 0.6104 on weekdays and *p* = 0.6897 at weekends). During weeks 11–18 (Lock), a significant reduction of IR is observed during weekdays (mean: from 93.23% in 2019 to 92.34% in 2020), but not during weekends (*p* = 0.8072). Moreover, this decrease in IR is also found during the post-lockdown week both on weekdays (mean: from 93.18% in 2019 to 92.68% in 2020) and at weekends (mean: from 92.88% in 2019 to 92.25% in 2020). Finally, the dispersion of the IR distributions is quite similar between both years during the lockdown and post-lockdown, despite showing a slight decrease in 2020. Hence, the variability of IR in Rome suburban acoustic environments is almost kept compared to the pre-lockdown, contrary to what was observed in Milan.

### B. Hour-per-weekly–based differences of ANEs

In order to study the temporal evolution along the day of the previously observed ANEs and salient events that drive the IR indicator, this section presents the analysis of the hour-per-weekly–based difference of their distribution between 2020 and 2019 from January to June for both urban and suburban acoustic environments. For each day, after computing the mean percentage of ANEs detected per hour [see Eq. (3)], this value is subsequently accumulated and averaged per weekdays or weekend days to obtain the corresponding $\Delta ANE^{h}$ (%) between 2020 and 2019. The difference between the presence of ANEs during the daytime and at night is also evaluated in terms of the mean ANE (%) within these time periods.

$$\Delta ANE^{h}=\frac{1}{N^{h,d}}\sum \left(ANE_{2020}^{h,d}-ANE_{2019}^{h,d}\right),$$ (3)

where $ANE_{2020}^{h,d}$ and $ANE_{2019}^{h,d}$ represent the percentage of ANEs detected by the ANED at hour *h* of day *d* of each year in all sensors per area, and $N^{h,d}$ stands for the total number of samples considered for the computation.

Moreover, as a by-product of the IR computation, the percentage of time that the $L_{\mathrm{Aeq},T}$ surpasses the 3 dB threshold is also computed (Brambilla *et al.*, 2020), obtaining the temporal distribution of salient events [hereafter denoted as Salients (%)] that can come either from ANEs (e.g., a siren) or RTN (e.g., a vehicle pass-by). Their evolution allows contextualizing the results obtained from the ANE analyses.

#### 1. Milan urban area

**FIG. 3.**
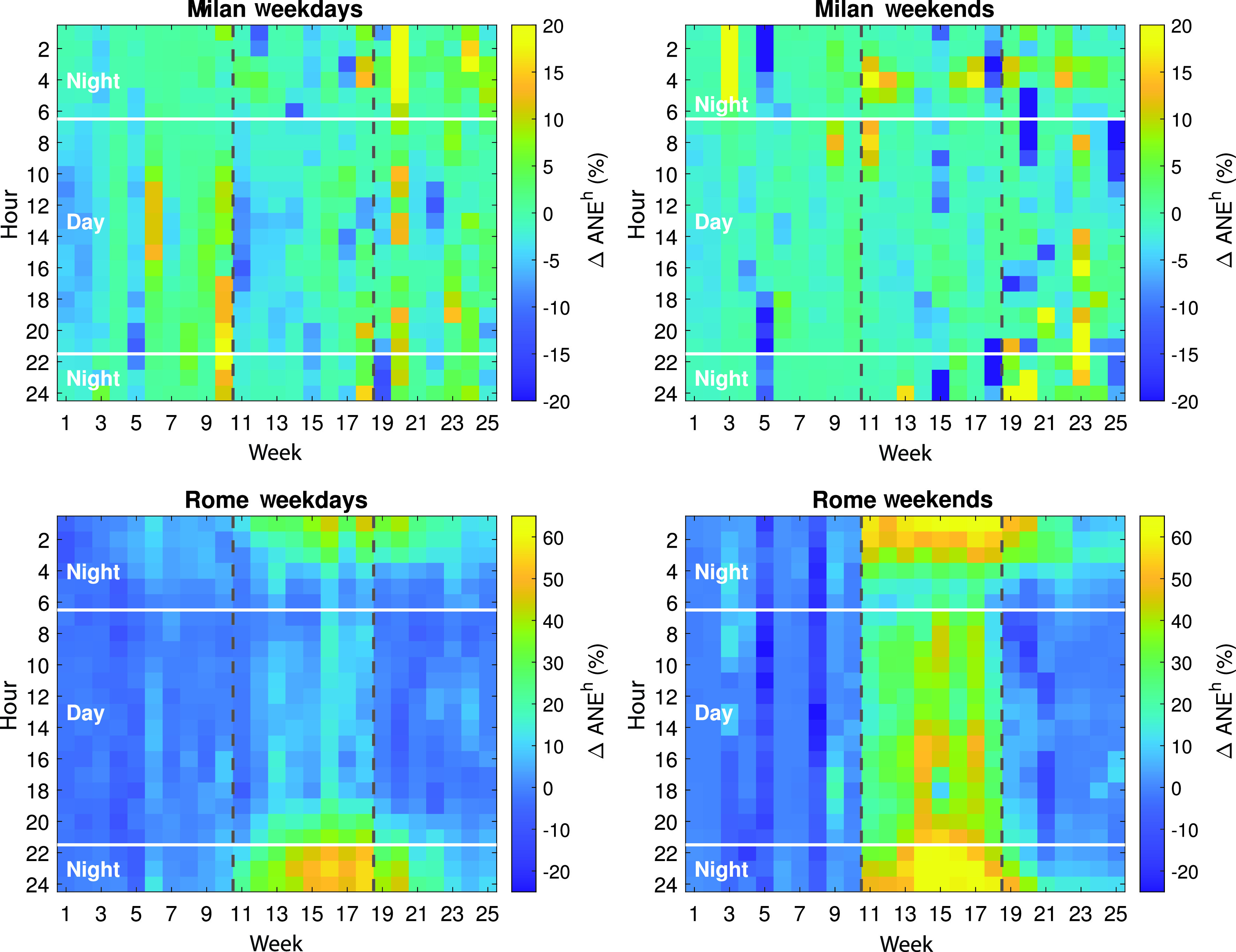
(Color online) Hour-per-weekly–based distribution of $\Delta ANE^{h}$(%) from January to June (weeks 1–25) in Milan and Rome urban and suburban areas during weekdays and weekends. The vertical dashed black lines represent the beginning and end of the lockdown (weeks 11–18), while the white horizontal lines represent the 6:00 h to 22:00 h day-night boundaries.

Figures 3(a) and 3(b) depict the hour-per-week $\Delta ANE^{h}$ (%) on weekdays and weekend days in the urban area, respectively. It can be observed that the reduction of ANEs during the lockdown is seen throughout the day, whereas the increase during the post-lockdown is mainly seen at night. Moreover, week 10 presents a significant increment of ANEs, mostly at afternoon and evening. This is the last week before the Italian authorities decreed the hard lockdown after several previous measures that caused a *panic effect* on Milan's population, as already highlighted in Zambon *et al.* (2021), where it was observed that this “*fear-of-running-out-of-pantry*” episode also caused an increase in the *L_Aeq_* levels.

This overall pattern analysis is complemented with the computation of the mean differences regarding the presence of ANEs and Salients in 2020 with respect to 2019, during the diurnal and nocturnal periods in Table II. The values shown in the left-most part of the table show that the previously stated reduction of ANE (%) during the lockdown is seen both on weekdays and weekends, but it is mainly due to variations in diurnal periods (mean: −2.01% on weekdays and −1.76% at weekends). In the post-lockdown stage, the aforementioned increment of ANEs is obtained on weekdays, and mostly at night. A similar pattern is also observed for Salients (%) in relative terms, despite their higher mean percentages (their mean decreases −13.24% on weekdays and −14.48% at weekends).

#### 2. Rome suburban area

The Rome suburban acoustic environment shows a smoother hour-per-weekly–based distribution of ANEs than the urban counterpart [see Figs. 3(c) and 3(d)]. Both weekdays and weekends present higher variations of $\Delta ANE^{h}$ (%) within the lockdown, which are also noteworthy during the initial phase of the post-lockdown (e.g., weeks 19 and 20), which are also noteworthy increments. Regarding their hourly based distribution, there is a greater presence of ANEs along the day at weekends than on weekdays, where the increments are mainly found at night. Both during the lockdown and post-lockdown periods, higher increments of ANEs can be found between 22:00 h and 05:00 h. Moreover, notice that the increase in the presence of ANEs according to the ANED labels can be also clearly observed along the day on weekends.

The rightmost part of Table II presents the *ANE^h^* (%) and Salients (%) and their corresponding Δ increments in Rome for the three periods of interest. Regarding the variations between day and night, although the increase in ANEs is observed during the lockdown on both weekdays and weekends, weekends more than double the values observed on labour days both during daytime and at night. The Δ Salients present an opposite pattern, as they significantly decrease both during the day and at night, showing higher reductions during the daytime, despite also presenting higher drops during weekends than on weekdays (almost doubling the values). During the post-lockdown, values at night remain high for ANEs and low for Salients, but they start to return to pre-lockdown *normality*.

## V. CONCLUSIONS

The conducted analyses show the different nature of both Milan urban and Rome suburban acoustic environments in what concerns the presence of ANEs and IR metric. In Milan, the ANED detects a lower percentage of ANEs than in Rome for all the evaluated periods; a result consistent with the lower presence of salient events. The mean ANE (%) distributions are significantly lower than those from the corresponding distribution of Salients (%). On average, ANEs only represent the 13% of Salients in the pre-lockdown both in Milan and in Rome, increasing to 18% and 55% during the lockdown, and returning to 28% and 21% in the post-lockdown, respectively. This result confirms that the ANED does not only detect very salient events from the background noise, being capable of discarding those coming from RTN.

After validating that both the distribution of ANEs and IR values in 2020 is equivalent to what was observed in 2019 during the pre-lockdown stage for environments, the conducted analyses show a statistically significant increment of ANEs in Rome with respect to 2019 (+24.9% on average), mainly on weekends (+35.6% on average), being especially relevant at night (+37.5% on average). This pattern is kept during the post-lockdown, in which ANE percentages start to return to pre-lockdown values, but still remaining higher (+5.8% on average), and again, especially at night (+12.3% on average). In Milan, a different behaviour was found, as one of the main sources of ANEs in the urban area is citizenship (Alías *et al.*, 2020b). ANE (%) significantly decreased during the lockdown (−1.4% on average) when citizens stayed at home (also observed in salient events with a mean reduction of 11%); a reduction mainly was observed during the day (−1.9% on average) as well as in salient events (−13.9% on average). This result is especially relevant considering the overall *L_Aeq_* reduction of 7 dB in Milan (Alsina-Pagès *et al.*, 2020; Zambon *et al.*, 2021), as lower RTN levels should allow the ANED algorithm to detect low and medium acoustically salient ANEs (Alías *et al.*, 2020a). The post-lockdown period shows slightly higher values than in 2019. Finally, notice that despite punctual peaks in ANE, hour-per-week differences were found in this city, probably due to meteorological episodes. According to ARPA, they are almost negligible as less than 5% of the days contain relevant rainfalls. Moreover, despite taking the 2020 pre-lockdown period, as well as 2019 distributions as baseline of the lockdown and post-lockdown analyses, they could have been affected by several uncertainties, such as the evolution of background noise and its effect on the ANED performance.

The conducted experiments have extended the initial insights observed in Alsina-Pagès *et al.* (2020), confirming the general higher presence of ANEs on weekends and during nocturnal periods in Rome, as lower salient ANEs (Alías *et al.*, 2020a) can be detected due to the decrease in RTN levels (5–6 dB, on average) (Alsina-Pagès *et al.*, 2020). However, relevant differences have also been found. The mean values of the variations of ANE (%) between 2020 and 2019 are significantly larger than those previously observed. And in Milan, the reduction of ANEs (%) has been observed, not only on weekdays, but also at weekends during the lockdown.

Regarding the impact on population, the IR indicator also shows different behaviours in both cities. In Rome, no location presents a relevant variation (larger than 1%) of IR during the pre-lockdown, at least in terms of changes in noise perception, as shown in Brink *et al.* (2016). Seven of the 11 sensed locations show a relevant reduction of IR in the lockdown (5 during the weekends and 2 at weekends, respectively), whereas only one location shows a noteworthy increase on weekends. Again, Milan presents a different behaviour. Eleven of the 20 considered sites present a relevant increment of IR during the lockdown with respect to three (six on weekdays and 5 at weekends with respect to 1 location and 2 locations in the pre-lockdown, respectively). The post-lockdown presents a similar behaviour in both cities, but with lower differences than in the pre-lockdown. Therefore, we can conclude that, in general terms, mainly the lockdown and also the post-lockdown periods have entailed significant differences in terms of the ANEs distribution, besides yielding an improvement on people's well-being. However, several locations have shown an opposite performance.

Future work will be focused on extending the conducted analyses to keep evaluating the effects of COVID-19 on both Milan urban and Rome suburban acoustic environments, as the effects of the pandemic are unfortunately still present.
